# Effects of leucism on organ development and molecular mechanisms in Northern snakehead (*Channa argus*) beyond pigmentation alterations

**DOI:** 10.1038/s41598-023-46608-9

**Published:** 2023-11-11

**Authors:** Wei Fan, Yang He, Jian Su, Yang Feng, Ting Zhuo, Jun Wang, Xiaolei Jiao, Yu Luo, Jun Wu, Yi Geng

**Affiliations:** 1 College of Veterinary Medicine, Sichuan Agricultural University, Huimin Street No. 211, Wenjiang, 611130 Sichuan People’s Republic of China; 2 NeiJiang Academy of Agricultural Sciences, Neijiang, 641000 Sichuan People’s Republic of China; 3 Key Laboratory of Sichuan Province for Fishes Conservation and Utilization in the Upper Reaches of the Yangtze River/College of Life Sciences, Neijiang Normal University, Neijiang, 641000 Sichuan People’s Republic of China

**Keywords:** Developmental biology, Genetics, Molecular biology, Physiology, Systems biology

## Abstract

Leucism, a widespread occurrence observed in Northern snakehead (*Channa argus*), bestows a striking white jade-like body coloration upon affected individuals and has gained substantial popularity in commercial breeding. While the visible manifestation of leucism in snakeheads is primarily limited to body coloration, it is crucial to explore the potential influence of leucism on organ development and elucidate the underlying molecular mechanisms. Through a comparative analysis of growth differences, our study revealed that at 150 days post-fertilization, the white variety exhibited an 8.5% higher liver index and intestinal index, but experienced a 20% and 38% decreased in spleen index and renal interstitial index, respectively, suggesting an enlarged digestive area but relatively smaller immune tissues. Nonetheless, no significant differences were observed in the intestinal flora between the two varieties, suggesting the exclusion of any exogenous impacts from symbiotic flora on the growth and development of the white variety. Importantly, transcriptome analysis demonstrated that the white variety exhibited higher expression levels of innate immune genes. Furthermore, annotation of the gene sets expressed in the liver and spleen revealed 76 and 35 genes respectively, with the white variety displaying lower expression in genes associated with “Viral protein interaction with cytokine and cytokine receptor”, “Protein processing in endoplasmic reticulum”, and “TNF signaling pathway”, while exhibiting higher expression in “Estrogen signaling pathway”. Notably, three genes, namely *pcdhf 4*, *nlrc3 card 15-like*, and a *pol-like* were identified in both the liver and spleen, indicating their potential involvement in altering the development and innate immunity of the white variety. This study reveals the systemic impact of leucism that extends beyond mere pigmentation alterations, highlighting the prominent characteristics of this phenotype and providing a foundation for future molecular breeding programs aimed at enhancing this variety.

## Introduction

The Northern snakehead (*Channa argus*) belongs to the Perciformes order, Anabantoidei suborder, Channidae family, and the genus *Channa*. It is widely distributed in major water systems throughout China, characterized by its dark black body with irregular cloud-like black patches on each side^1, 2^. *C. argus* holds significant commercial value due to its high meat content, fewer intermuscular bones, and excellent meat quality compared to many freshwater fish species^3, 4^. In 2020, China's annual production of *C. argus* surpassed 500,000 tons, resulting in over one billion US dollars in direct economic benefits. Additionally, it has provided employment and economic opportunities for hundreds of thousands of farmers^5^. Building upon these achievements, efforts have been made to breed superior varieties of *C. argus*. For instance, the growth rate of YY super-male snakeheads, a breed developed through sex reversal and molecular sexing, has been enhanced by 17.3%^6^. Furthermore, hybrid snakeheads obtained by crossbreeding *Channa maculata* (female parent) and *C. argus* (male parent) have been explored^7^. On the other hand, one notable drawback of wild snakeheads and hybrid snakeheads is their resemblance to snakes, which might deter some of the customers. Therefore, the development of new snakehead varieties with altered physical appearances holds great potential for driving advancements in related industries.

**Figure 1:**
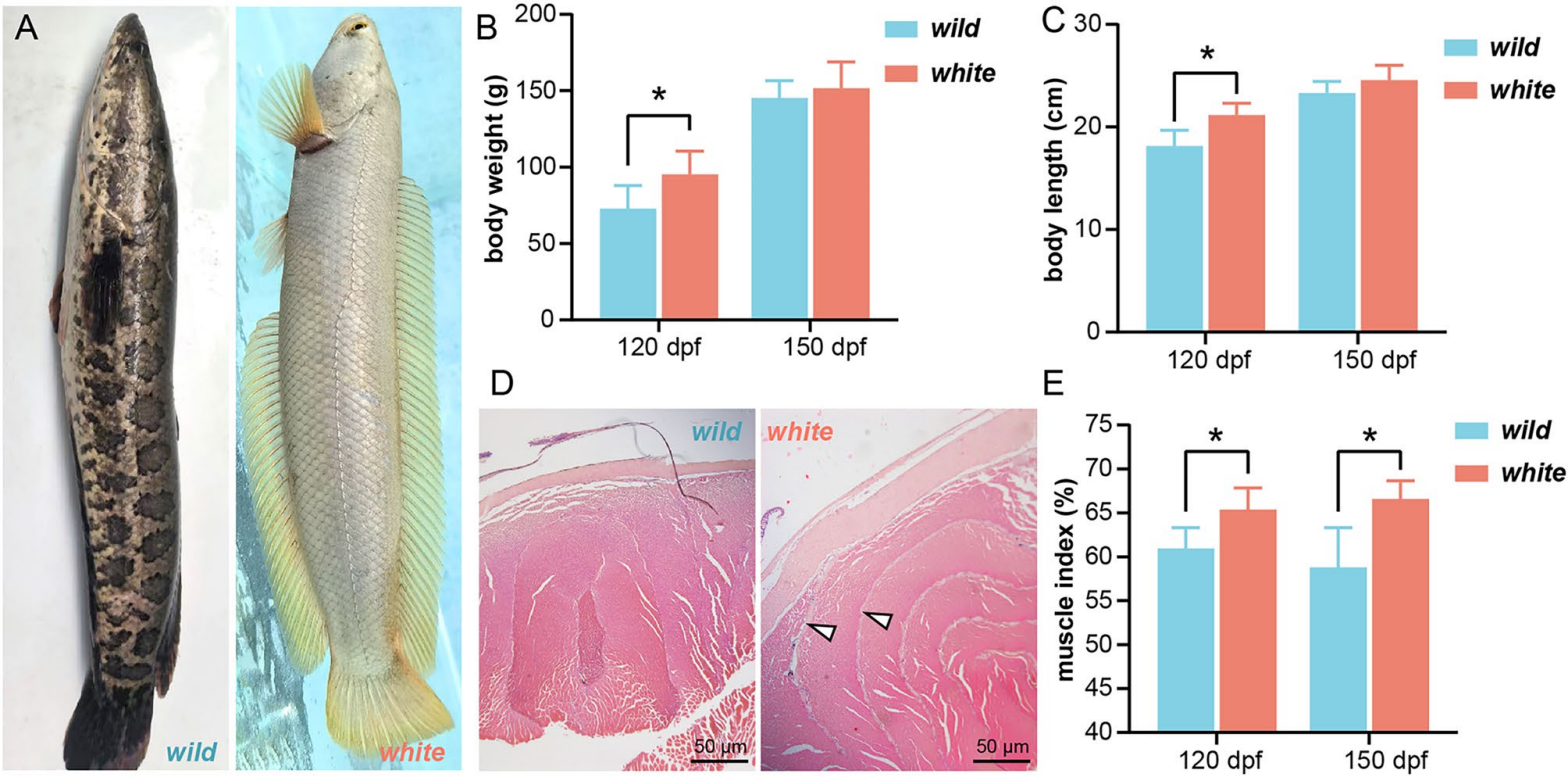
The comparison regarding the growth characteristics of white and wild *C. argus*. (**A**) The appearance of the two varieties, wild and white *C. argus*, was depicted. (**B**), (**C**) The variations in body weight and body length, respectively, between the two varieties at 120 dpf and 150 dpf (ANOVA, n = 10). (**D**) The histology of skeletal muscle at 150 dpf, specifically highlighting the perimysium, as indicated by the arrows, using H&E staining at a magnification of × 40. (**E**) The muscle index (muscle weight/body weight × 100%) between two varieties (ANOVA, n = 10). Notably, the asterisk (*) denotes a statistically significant difference (*P* < 0.05) observed between the two varieties.

Albinism / leucism are common phenomena observed in *C. argus* within the Jialing River system and Tuojiang River system in Sichuan Province, China. It serves as a significant method for eliminating the serpent-like appearance, wherein the synthesis of pigments within the organism's body is obstructed, consequently leading to the manifestation of a white, golden-white, or light-golden exterior form^8^. The emergence of the yellow-albino variety may be attributed to the potential involvement of genes associated with pathways such as MAPK, WNT, and calcium signaling, which potentially increase melanogenesis elements and are likely stimulated by fibroblast-derived melanogenic factors^9^. On the other hand, the production of white varieties may be due to the fact that melanin synthesis, WNT, and MAPK signaling pathways are associated with skin depigmentation in *C. argus*, and the low expression of pigment-related genes contributes to the manifestation of whitening characteristics^10^. Recently, a new white (leucism) variety of *C. argu*s*,* referred to as the “Chinese White Jade Dragon [variety registration number: GS-01-005-2022]” has gained popularity. This variety exhibits a snow-white body surface and yellow-gold fins, symbolizing wealth and prosperity, which has gradually entered the high-end food market (Fig. 1A)^11^. The white variety is sold at a price three times higher than the wild variety, providing greater economic benefits to farmers. Therefore, the development of white variety culture not only contributes to the conservation of *C. argus* diversity but also introduces novel and economically viable opportunities into traditional snakehead culture.

Building upon the observed morphological changes, the next stage in molecular breeding is to further extend the foundational traits of stable varieties, even amidst this process. Leucism, which gives rise to distinctive varieties, is a common occurrence in fish genetic variation^10^. Consequently, the selection and breeding of superior traits in white *C. argus* assume paramount importance. Notably, Zhou et al.^12^ discovered that the white variety exhibits a higher N-3 to N-6 polyunsaturated fatty acids, including EPA+DHA, compared to the wild variety. Additionally, Wang et al.^13^ demonstrated that the white variety possesses higher protein content and lower fat content in its body composition. These findings provide valuable breeding implications for white *C. argus*. However, the development disparities and molecular mechanisms underlying selective breeding to enhance the growth performance of the white variety remain unclear. Thus, it is crucial to investigate the potential influence of leucism on organ development and elucidate the underlying molecular mechanisms. The sequencing and gradual refinement of the *C. argus* genome offer a more reliable reference for investigating these molecular mechanisms^14^. Currently, transcriptomic sequencing has been conducted on a substantial number of relevant samples^15^, encompassing diverse areas such as infection^16^, toxicology^17^, and genetic development^18^. Moreover, the construction of a genetic linkage map has enabled the creation of a comprehensive sex-averaged map and sex-specific genetic maps for the *C. argus*^19, 20^. In contrast, when it comes to the leucism variety of the *C. argus*, transcriptomic investigations have predominantly focused on unraveling the mechanisms underlying skin depigmentation^10^. However, there remains a noticeable dearth of studies examining the regulation of organ growth differences in leucism variants. This knowledge gap poses challenges to the advancement of selective breeding programs for this particular variety. This study aims to compare the growth discrepancies between the white and wild varieties during different growth stages, identify specific growth-related genes and regulatory mechanisms, and establish a foundation for prospective molecular breeding programs aimed at enhancing this variety. Simultaneously, it seeks to uncover the comprehensive impact of leucism beyond its cutaneous manifestations.

## Results

### Comparative analysis of early development and growth characteristics in white and wild *C. argus*

In this study, we aimed to investigate the early developmental differences between two varieties of *C. argus* under the same aquaculture environment and management conditions. The distinct body coloration allowed for easy differentiation of the two varieties, with the white variety showcasing a remarkable appearance, characterized by golden fins and white jade scales (Fig. 1A). Prior to each sampling event, the fish from both varieties were weighed, and the results revealed a noteworthy dissimilarity in the growth rates during the initial developmental stages. Specifically, our examination of the white variety indicated a significantly faster growth rate compared to the wild variety (Fig. 1B,C). Nonetheless, this growth advantage gradually diminished over time, as both varieties exhibited similar growth rates at 150 days post-fertilization (dpf) (Fig. 1B,C). No statistically significant disparity in muscle fiber was detected between the two varieties; however, it was noted that the white variety exhibited a relatively thicker perimysium (Fig. 1D). When comparing the harvested edible muscle portions, the white variety exhibited a significantly higher muscle index at 120 dpf and 150 dpf compared to the wild one (Fig. 1E), implying that white *C. argus* potentially possesses a greater yield of edible meat.

### Comparative analysis of organ development and variation in white and wild varieties

**Figure 2:**
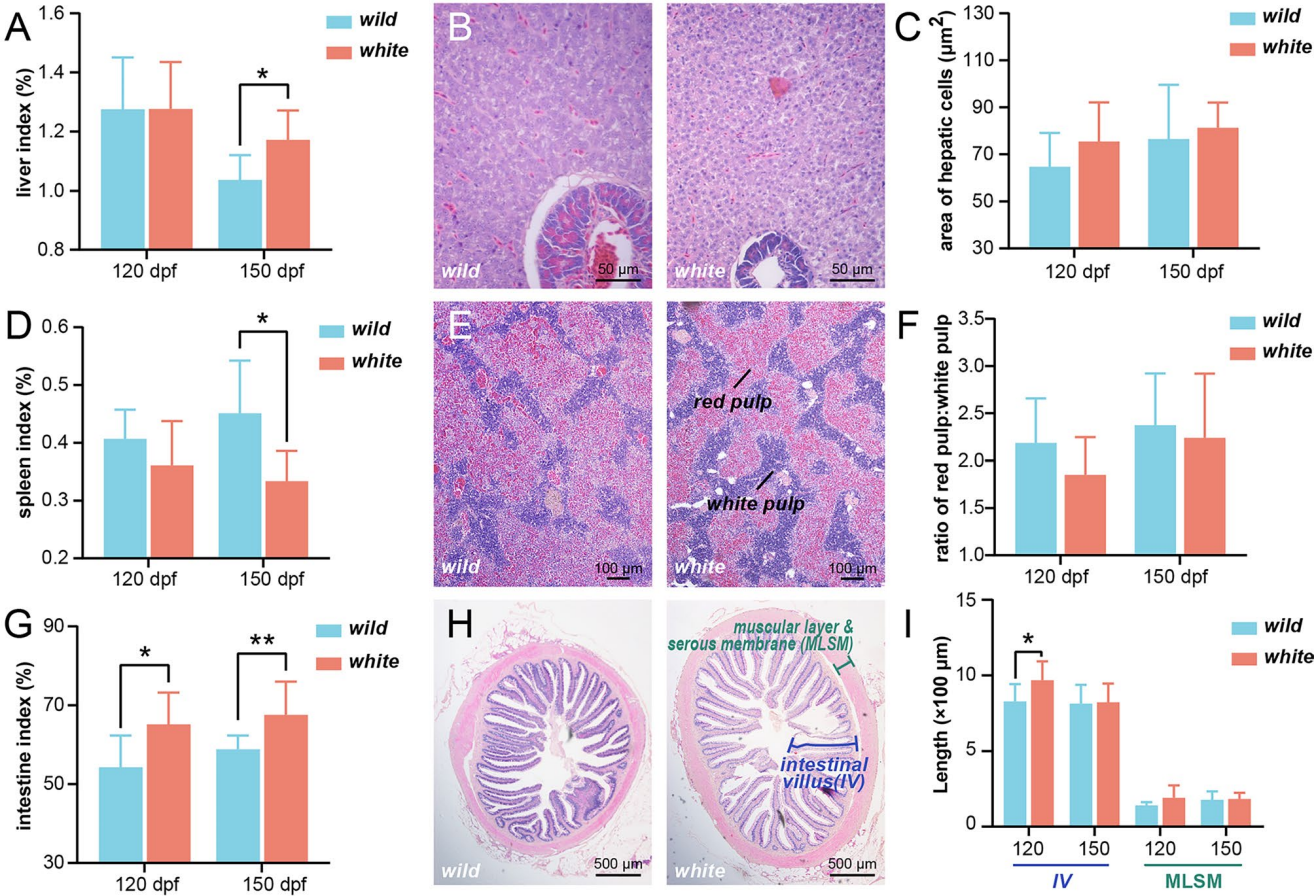
The analysis of organ indices and histological measurements in white and wild *C. argus*. (**A**), (**D**), & (**G**) The liver index, spleen index, and intestine index (organ weight/body weight × 100%) were examined for both white and wild *C. argus*, respectively (ANOVA, n = 10). (**B**), (**E**), & (**H**) The histology of liver (× 400), spleen (× 100), and intestine (× 40) was assessed through H&E staining in white and wild *C. argus*, respectively (n = 10). (**C**) The area of hepatic cells in the liver was measured and compared between the two varieties at 120 dpf and 150 dpf (ANOVA, n = 10). The average area of each liver cell in an individual is determined by dividing the area of liver tissue in the slice (excluding tissues like the pancreas and blood vessels) by the number of cell nuclei. (**F**) The ratio of red pulp to white pulp in the spleen was determined for both varieties at 120 dpf and 150 dpf (ANOVA, n = 10). The relevant ratio was obtained by dividing the area of the red pulp by the area of the white pulp. (**I**) The length of intestinal villus (IV) and muscular layer & serous membrane (MLSM) in the intestine was determined for both varieties at 120 dpf and 150 dpf (ANOVA, n = 10). All evaluations were performed using Image Processing and Analysis in Java (https://imagej.net/ij/), with the calculation rules for the corresponding area or length documented in the figure. Statistical significance was indicated by *, representing a significant difference (*P* < 0.05), or **, representing a highly significant difference (*P* < 0.01), between the two varieties.

Then, we have conducted comprehensive measurements and histological analyses to compare the organ indexes, coloration, and tissue structures of various organs such as the liver, spleen, kidney, heart, intestine, and gills in order to assess their developmental differences. Remarkably, notable variations were observed in two varieties in the indexes of liver, spleen, kidney, and intestinal at 150 dpf. Specifically, we found that the proportions of liver and intestinal indexes were significantly increased in the white variety (Fig. 2A,G), while the proportions of spleen and kidney indexes were significantly decreased compared to the wild variety (Fig. 2D, S1A). Furthermore, our organ color analysis revealed a significant paleness of the liver (see Supplementary Fig. S1E online), whereas no significant change was observed in the color of the spleen and kidney (see Supplementary Fig. S1B,F online). Histological studies of the liver showed normal morphology in both varieties at 120 dpf, with moderate granular degeneration observed at 150 dpf, but no significant difference was observed among different varieties (Fig. 2B,C). However, the area of the white pulp in the white variety was slightly decreased compared to the wild variety, particularly noticeable at 120 dpf, although the difference was not statistically significant (Fig. 2E,F). In contrast, the length of intestinal villus in the white variety was significantly higher than that of the wild variety at 120 dpf (Fig. 2H,I). Based on these findings, it appears that the white variety exhibits a bigger digestive system, which may contribute to its higher muscle index. Additionally, our observations implied that the white variety displays relatively weaker immune capacity. This was also suggested by renal histology, where the renal interstitial ratio of the white variety was lower than that of the wild variety (see Supplementary Fig. S1C,D online), indicating a relatively low hematopoietic immune function. Furthermore, no significant difference was observed in gills and heart indexes (see Supplementary Fig. S1G,H online), indicating similar respiration and pumping capacity in both varieties.

### Microbiota composition and developmental differences in two *C. argus* varieties

**Figure 3:**
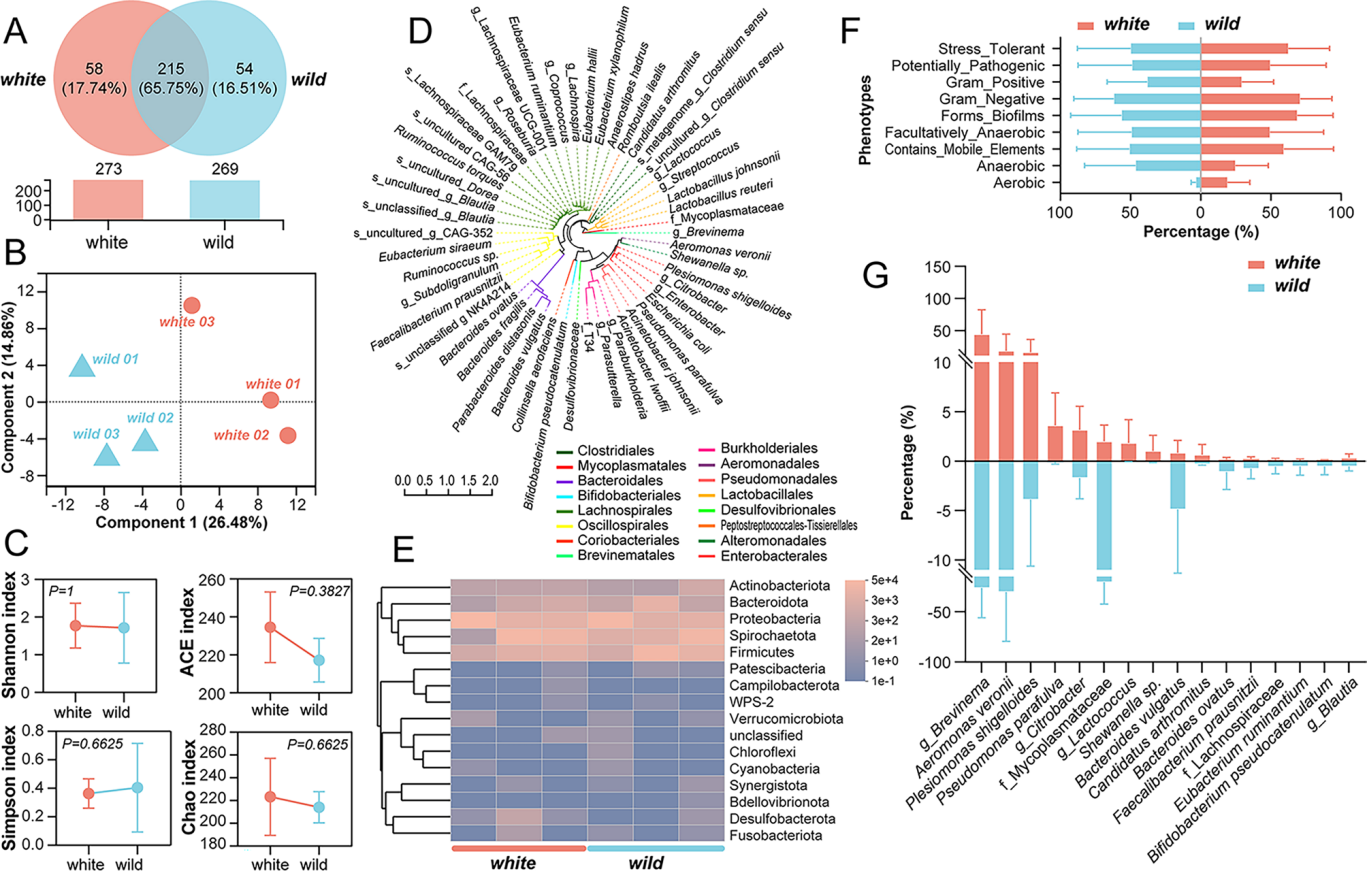
The comparative analysis of the intestinal microbial community between two varieties. (**A**) Venn diagram illustrating the species overlap between the two varieties. (**B**) Partial Least Squares Discriminant Analysis (PLS-DA) plot depicting the distribution of samples from the two varieties. (**C**) Results of the alpha diversity test includes Shannon index, ACE index, Simpson index, and Chao index, assessing the diversity within each variety (*t*-tests, n = 3). (**D**) Phylogenetic tree displaying the bacterial species present in variety *C. argus*. (**E**) Relative abundance of different phyla in the two varieties (*t*-tests, n = 3). (**F**) Bugbase phenotype annotation, providing information on the phenotypic characteristics of the microbial species (*t*-tests, n = 3). (**G**) Distribution of the major bacterial taxa across different intestinal flora (*t*-tests, n = 3).

The host provides an opportunity to bacteria for a symbiotic relationship^21, 22^, which are involved in regulating internal satiety signals, digestion and absorption, neural pathways, immune pathways, and cytokines^23^. Thus, the magnitude of organism growth is hypothesized to be influenced by a combination of exogenous and endogenous genes, with the exogenous genetic elements potentially being regulated by the symbiotic bacterial populations residing within the host organism. In order to investigate this phenomenon, we extracted DNA from the contents of the mid-intestine to hindgut of two varieties of *C. argus*, and compared their intestinal microbiota composition using 16S-seq. A total of 110,572,855 valid sequences, with an average length of 426 bp, were obtained, as shown in Table 1. Annotation analysis identified 327 species (similarity: < 97%) across all samples, belonging to 216 genera and 16 phyla. Among these, 215 species were common to both varieties, while 58 and 54 species were exclusively annotated in the white and wild *C. argus*, respectively (Fig. 3A). Notably, samples from both varieties did not exhibit distinct clustering according to the PLS-DA analysis (Fig. 3B). Additionally, the diversity test revealed no significant difference in community diversity (Fig. 3C). The predominant bacteria found in the intestinal tracts of *C. argus* included Actinobacteriota, Bacteroidota, Proteobacteria, Spirochaetota, and Firmicutes (Fig. 3D), with no significant differences observed between the two varieties (Fig. 3E). These findings suggest that, within the same aquaculture environment, the white variety of *C. argus* did not exhibit distinct bacterial selection preferences compared to the wild variety. Furthermore, this indirectly indicates the negligible impact of the exogenous gene set, composed of symbiotic microbial communities, on the developmental differences between the two varieties. However, phenotypic analysis indicated that the white variety demonstrated a higher aerobic capacity compared to the wild variety, while this distinction did not exhibit statistically significant differences (*P* > 0.05) (Fig. 3F). Analysis of the larger species distribution within the two groups revealed that both varieties carried a high proportion of *Aeromonas veronii*, while variations were observed between the two varieties in terms of *Plesiomonas shigelloides*, family Mycoplasmataceae, *Pseudomonas parafulva*, and genus *Shewanella* (Fig. 3G). Despite these discernible variations, statistical analysis revealed a lack of significant disparities (*P* > 0.05).

**Table 1:** Total quality of *16S-seq* samples.

Sample Info	Seq_num	Base_num	Mean_length	Min_length	Max_length
Wild 01	47,257	19,989,579	422.99	338	430
Wild 02	40,885	17,520,950	428.54	403	430
Wild 03	40,379	17,237,948	426.90	402	430
White 01	34,600	14,739,228	425.99	388	430
White 02	46,184	19,777,775	428.24	403	430
White 03	49,700	21,307,375	428.72	392	430

### Unraveling the transcriptomic profile of selected organs in two varieties of *C. argus*

In order to explore the genetic differences underlying organ development induced by the host, we extracted mRNA from the livers and spleens of two varieties to construct cDNA libraries. The Illumina Novaseq 6000 platform was utilized for the sequencing process. Following the completion of data generation and quality control protocols, the Clean Data of all samples exceeded 6.16 Gb, with a Q30 base percentage of over 94.31%. Subsequently, the clean reads of the samples were aligned to the reference genome GCA_004786185.1 using RSEM (utilizing Bowtie 2 with a parameter mismatch of 0) for mapping analysis. The mapping rate of each sample was 90.29% ± 5.76%, which met the requirements for RNA-seq analysis utilizing a reference genome (Table 2). Data analysis was performed using Trinity, and annotation was conducted using Clusters of Orthologous Groups (COG), Gene Ontology (GO), Kyoto Encyclopedia of Genes and Genomes (KEGG), Clusters of orthologous groups for eukaryotic complete genomes (KOG), Pfam, and Swiss-prot databases, resulting in the identification of 23,966 genes, including 17,048 known genes and 6,918 novel genes.

**Table 2:** Total quality of RNA samples.

Variety	Samples	Raw reads	Clean reads	Error rate (%)	Q30 (%)	GC content (%)	Mapping rate (%)
Wild	liver-01	47,591,352	46,883,618	0.0242	94.95	47.34	89.25
	liver-02	42,841,296	42,310,742	0.024	95.15	46.34	94.9
	liver-03	45,328,082	44,666,082	0.0242	94.98	47.1	88.61
	Spleen-01	44,345,952	43,400,192	0.0247	94.45	47.05	93.64
	Spleen-02	45,892,792	43,879,064	0.0245	94.69	47.79	94.2
	Spleen-03	44,831,064	43,763,322	0.0249	94.31	44.14	93.33
White	liver-01	56,849,010	56,092,196	0.0238	95.4	47.18	94.38
	liver-02	44,598,440	43,999,860	0.0238	95.35	46.92	91.88
	liver-03	48,637,954	47,975,682	0.0238	95.38	46.96	92.27
	Spleen-01	44,674,788	43,748,714	0.0246	94.63	46.21	81.73
	Spleen-02	43,521,044	42,252,280	0.0243	94.86	48.42	76.17
	Spleen-03	44,799,814	43,894,616	0.0248	94.37	43.64	93.1

**Figure 4:**
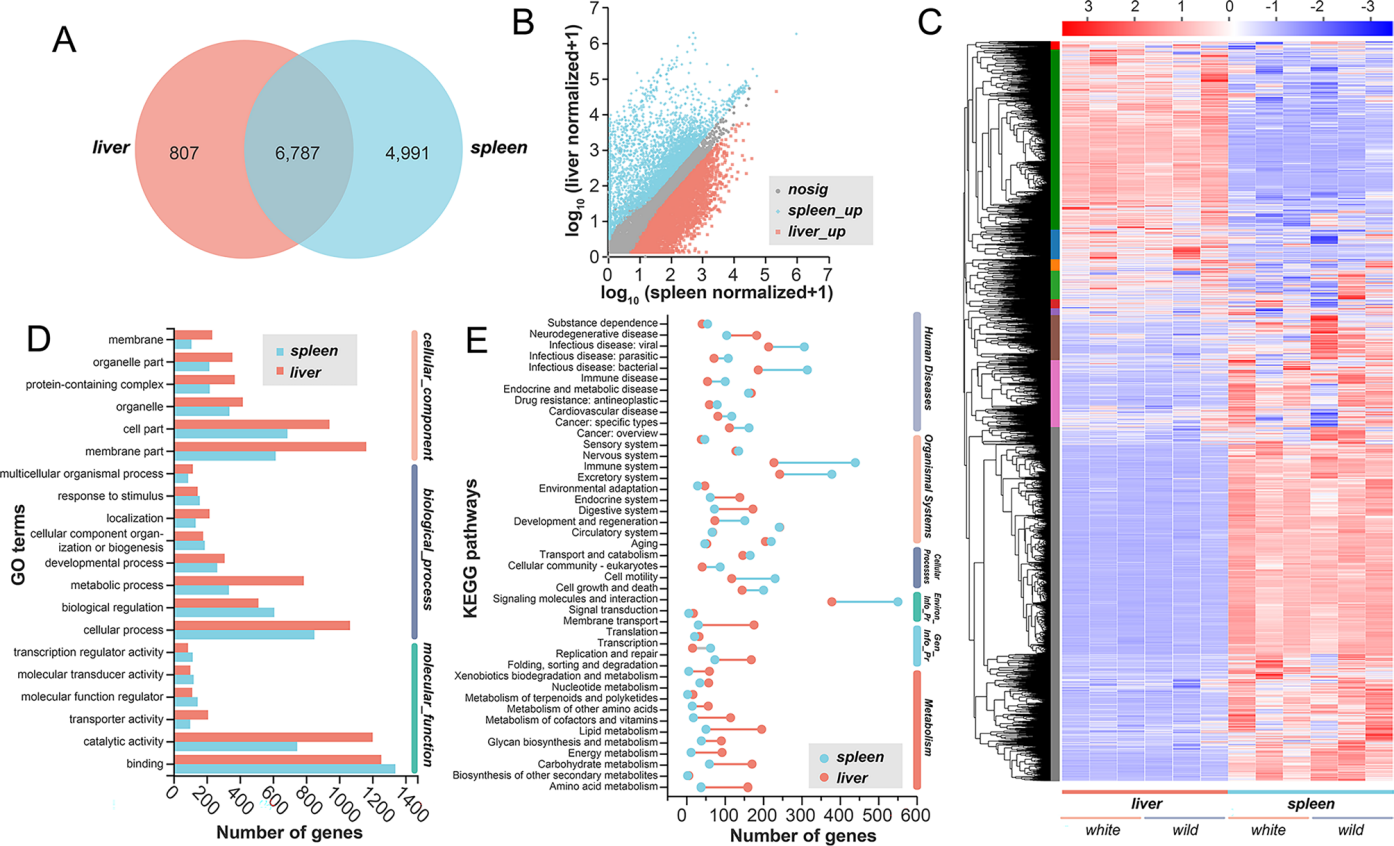
Comparative analysis of the liver and spleen transcriptomes identifies differentially expressed genes. (**A**) Venn diagram illustrating the overlap between the liver and spleen. (**B**) Scatter plot displaying the expression differences between the two organs. (**C**) Heat map depicting the differential expression in the liver and spleen. (**D**) Gene Ontology (GO) annotation of highly expressed genes in the spleen and liver^24^. (**E**) Kyoto Encyclopedia of Genes and Genomes (KEGG) annotation of highly expressed genes in the spleen and liver^25^. n = 3.

Firstly, disregarding the specific varieties, we employed the DESeq2 algorithm with a screening threshold of |log2FC|> = 1 and *P* adjust < 0.05 to carry out differential gene analysis between two organs, aiming to obtain the transcriptome profiles related to organ development in *C. argus*. Consequently, we identified a total of 6787 genes that were commonly expressed, whereas 5798 genes exhibited differential expression in the spleen and liver. Notably, among these differentially expressed genes, 807 genes displayed higher expression levels in the liver, while 4991 genes exhibited elevated expression levels in the spleen (Fig. 4A–C). The GO enrichment analysis indicated that the differentially expressed genes (DEGs) were involved in Cellular components, Biological processes, and Molecular functions, exhibiting minimal disparity between the two organs (Fig. 4D). According to KEGG results, highly expressed genes in the liver were predominantly associated with Metabolism and Gene information processing, whereas highly expressed genes in the spleen were mainly linked to Human diseases, Environmental information processing, and pathways related to immunity and the excretory system (Fig. 4E).

### Identification of DEGs in the liver and spleen in two *C. argus* varieties

**Figure 5:**
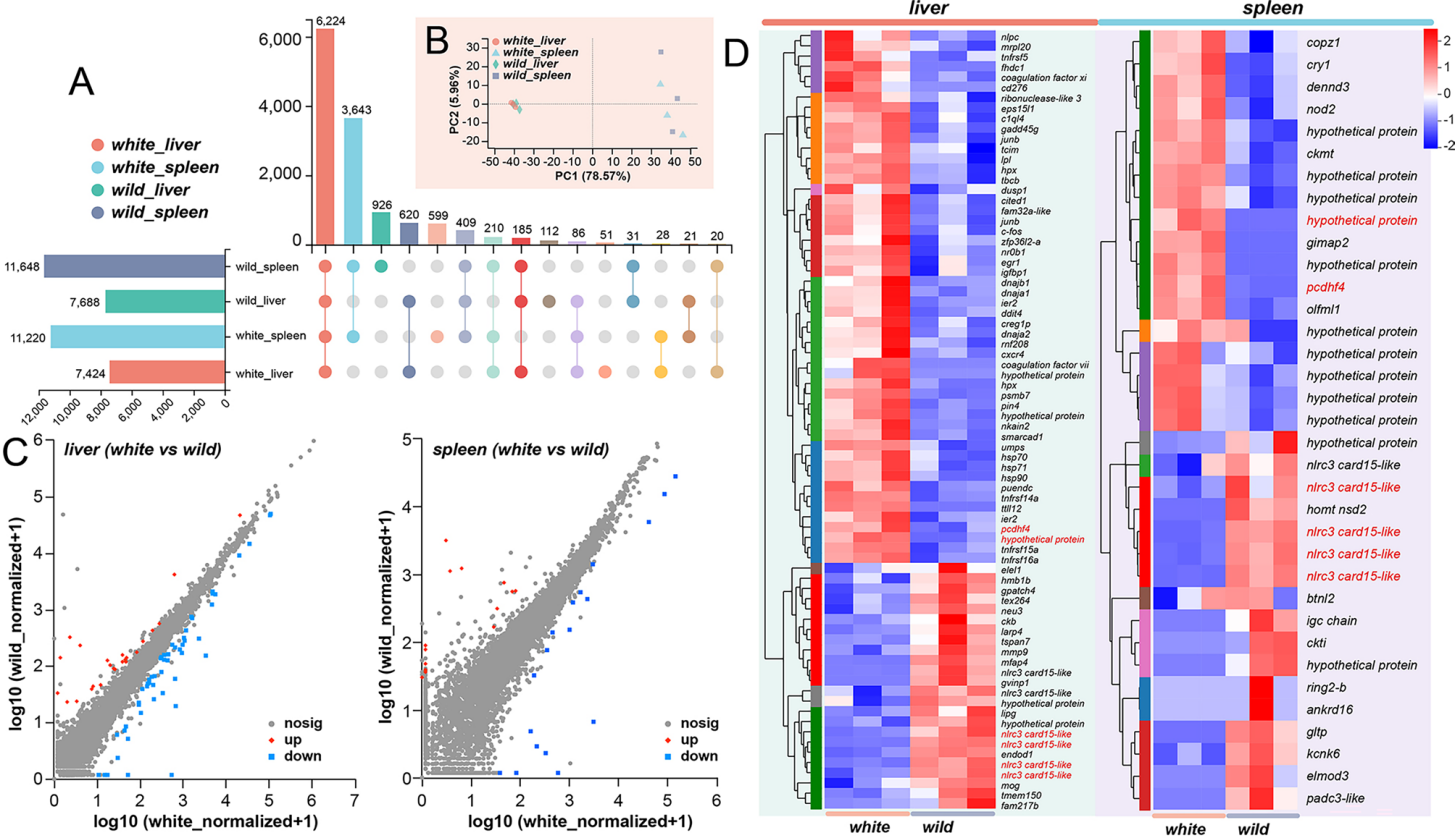
Comparative analysis identified gene expression profiles in the liver and spleen of different varieties. (**A**) Upset diagram illustrating the distribution of gene expression patterns in the liver and spleen between different varieties. (**B**) Principal components analysis (PCA) plot showing the clustering of samples based on gene expression profiles in the liver and spleen. (**C**) Scatter plot depicting the differential expression of genes between the liver and spleen in different varieties. (**D**) Heat map representing the differential expression of genes in the liver and spleen between different varieties (Deseq2 & BH method). Genes marked with red indicate their presence in both the liver and spleen. n = 3.

Then, taking into account the expression differences between the two varieties in the respective organs, we performed separate comparative analyses of gene expression in the liver and spleen using the DESeq2 algorithm. Our investigation unveiled a substantial number of expressed genes, with over 7000 genes in the liver and more than 10,000 genes in the spleen (Fig. 5A). Interestingly, a total of 6224 genes were found to be expressed in two organs examined (Fig. 5A). Despite the similarities observed in the overall expression patterns between the liver and spleen of both wild and white *C. argus* varieties (Fig. 5B), we identified 76 and 35 DEGs in the liver and spleen, respectively (Fig. 5C, see Supplementary Table 1 online). Notably, we found that the genes *pcdhf 4*, *nlrc3 card 15-like*, and a hypothetical protein gene containing a DNA/RNA polymerases domain (*pol-like*) were found to be differentially expressed both in the liver and spleen transcriptomes (Fig. 5D). Interestingly, *nlrc3 card 15-like* exhibited down-regulation, whereas *pcdhf 4* and *pol-like* showed up-regulation in white variety compared to the wild variety.

### Enrichment analysis of DEGs in the liver and spleen in two *C. argus* varieties

**Figure 6:**
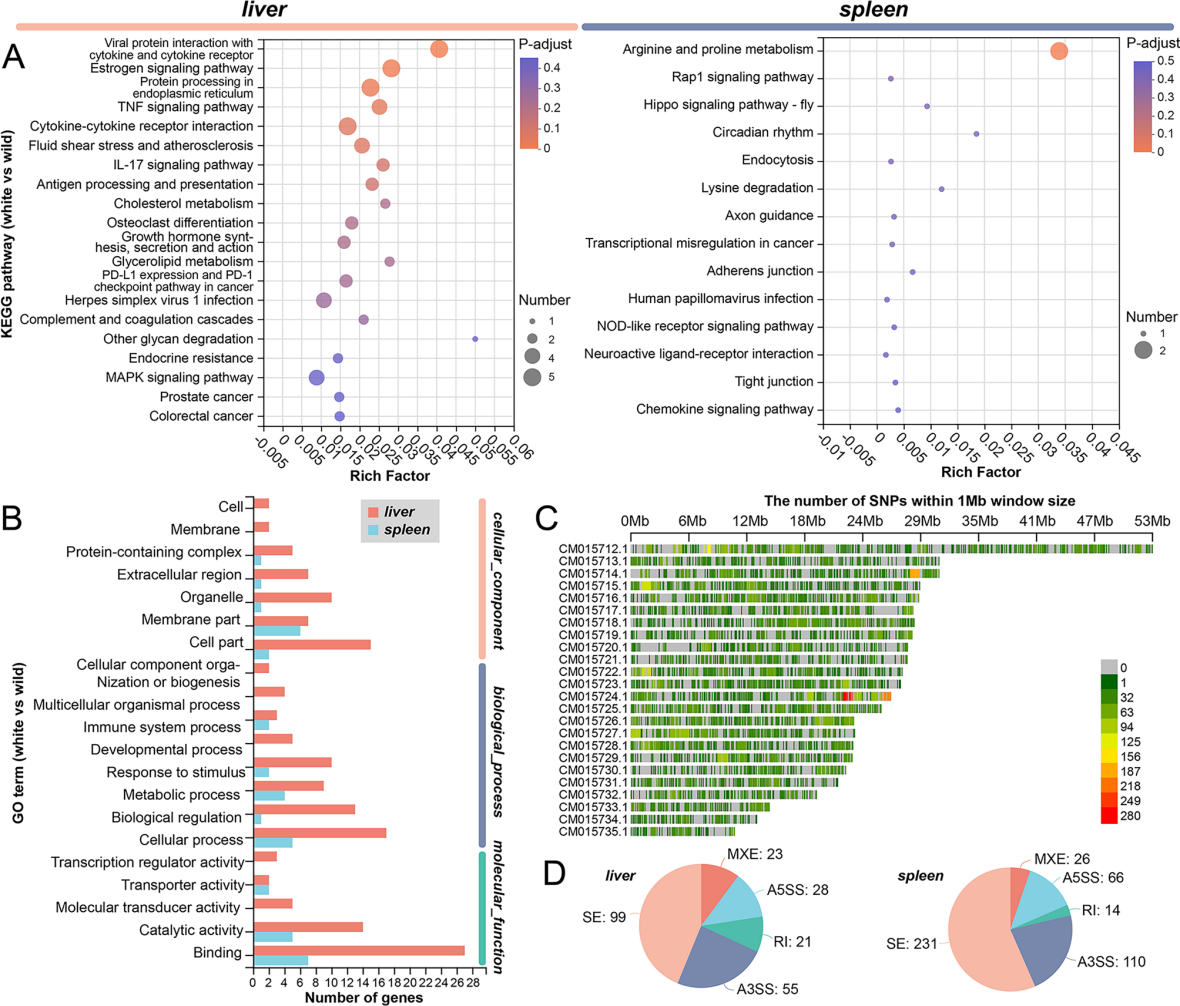
Gene function annotation and structural variability analysis of liver and spleen in different varieties. (**A**) KEGG enrichment analysis of differentially expressed genes (DEGs) in liver and spleen^25^. (**B**) Gene Ontology (GO) annotation of DEGs in liver and spleen^24^. (**C**) SNP/InDel analysis comparing different varieties. (**D**) Analysis of alternative splicing in liver and spleen between varieties.

Furthermore, our analysis of DEGs allowed us to identify significant pathways enriched in the liver and spleen. In the liver, we observed notable enrichment in pathways associated with Viral protein interaction with cytokine and cytokine receptor (VPIWC&CR), Estrogen signaling (ESP), Protein processing in the endoplasmic reticulum (PPIER), TNF signaling (TNFSP), and Cytokine-cytokine receptor interaction (CCRI) (Fig. 6A). Conversely, in the spleen, the primary enriched pathway was found to be related to Arginine and proline metabolism (A&PM) (Fig. 6A). Additionally, through gene ontology (GO) analysis, we discovered specific pathways concentrated in Anatomical structure development in the liver and Phosphorus metabolic process in the spleen (Fig. 6B, see Supplementary Figs. S2 and S3 online). Lastly, we conducted a thorough analysis of the transcriptome data, which led to the identification of numerous single nucleotide polymorphisms (SNP/InDel) differences predominantly located on chromosome CM015724.1 in the white variety (Fig. 6C). Moreover, we observed significant alternative splicing events in both the liver (226) and spleen (447) (Fig. 6D).

## Discussion

Despite the observed pigmentation alterations associated with leucism in snakeheads, the precise molecular mechanisms underlying the enhanced growth performance of the white variety remain poorly understood. In this study, we sought to elucidate the biological factors contributing to this phenomenon. Our findings reveal that the white variety exhibits a relative higher growth rate, increased muscle index, and improved digestive area compared to the wild variety, all while displaying a relatively smaller immune tissues under identical feeding conditions. To gain further insights, we constructed a comprehensive transcriptional map of the liver and spleen of *C. argus* and conducted a thorough characterization of the gene expression profiles within these organs. Through transcriptome analysis, we discovered numerous SNP/InDel events, predominantly located on chromosome CM015724.1. Furthermore, our annotation of liver and spleen genes unveiled 76 and 35 genes, respectively, displaying significant enrichment in pathways such as “VPIWC&CR”, “ESP”, “PPIER”, “TNFSP”, “CCRI”, and “A&PM”. These findings present potential molecular markers that can be utilized for the selection of the white variety. By extending our understanding of leucism beyond its superficial manifestations, this study highlights the remarkable traits associated with this phenotype and lays the groundwork for future molecular breeding programs aimed at further enhancing this variety.

Organs serve as fundamental entities governing animal life processes and act as the material foundation for their physiological functions. The Organ index, to some extent, provides insight into the organ workload within the organism^26^. Our research findings indicate noticeable variations in the liver and intestinal indexes, a reduction in the spleen and kidney indexes, while no significant disparity in gill and heart. These observations imply that the white variety has undergone specific modifications in its digestive and immune systems, while its respiratory and circulatory capacities remain unaffected. Of particular interest, the liver and intestinal indexes of the white variety exhibited a remarkable increase of 8.5%, with no discernible histological distinctions. This observation implies that, at an equivalent cellular functional level, the white variety possesses a relatively higher proportion of functional areas for digestion and detoxification during its early development, which could accelerate the growth rate for aquaculture. Moreover, we detected higher expression levels of “ESP” which may potentially contribute to enhanced cell growth, transcription, apoptosis inhibition, and pathogen interaction^27^. These factors significantly impact individual development. Additionally, the muscle index displayed a noteworthy 10.5% increment, reflecting an enhancement in developmental capability, and suggesting that the white variety possibly possesses improved physical agility. Importantly, this translates to substantial benefits for aquaculturists, as it yields more profitable outcomes, making it a cost-effective commodity for consumers.

According to the results, the host possesses the ability to influence the selection of specific bacterial communities through immunoglobulin (Ig) A or IgM^21, 22^. Based on the findings of this study, under the same rearing environment, no significant structural differences were observed between the microbial communities of the white and the wild varieties. This suggests that their selection ability remains unaffected by leucism. Considering the crucial role of intestinal microbiota in host growth and immune function, the unaltered colonization of these microbiota also largely eliminates potential impacts on the organ development of white variety. This helps to better explore potential genetic difference in the host itself. Nevertheless, at the genus level, the white variety exhibited higher levels of *P. shigelloides* and *P. parafulva* in its intestinal flora, which are opportunistic fish pathogens^28, 29^. Notably, the white variety showed relatively low levels of the family Mycoplasmataceae and Genus *Shewanella*, which are two common potential probiotic symbiotic bacteria in fish^30–32^. This indicates a certain risk in the symbiotic bacterial selection mechanism within the white variety. In combination with the immune tissue development of the white variety, this risk may be associated with the immune function's burden. Specifically, the white variety demonstrated significantly lower immune organ index, with the spleen and renal interstitial index decreasing by 20% and 38% respectively, at 150 dpf compared to the wild variety. Further analysis unveiled that the expression of "A&PM" was relatively high in the spleen of the white variety, which is involved in the biosynthesis of arginine and proline and has unclear effects on its immune capacity^33, 34^. Furthermore, decreased expression levels of “VPIWC&CR”^35^, “PPIER”^36^, “TNFSP”^37^, and “CCRI”^38^ were observed in the liver of the white variety. Therefore, overall, the white variety exhibits relatively low proportions of the spleen and renal interstitial tissues with no apparent compensatory pathways. This highlights the need for focused attention in future breeding efforts to seek germplasm optimization.

The concurrent annotation of *pcdhf4*, *nlrc3 card 15-like,* and *pol-like* in the liver and spleen suggests their potential role as significant modulated genes in response to leucism in these organs. The upregulation of *pcdhf4* and *pol-like*, along with the significant downregulation of *nlrc3 card 15-like*, is observed in the white variety. Notably, *pcdhf4*, an upstream regulatory protein of the Hippo signaling pathway^39^, governs crucial cellular processes such as cell proliferation, apoptosis, and fate^40^. Its loss can disrupt planar cell polarity and oriented cell division^41^. On the other hand, *pol-like* is an unverified gene, but its DNA/RNA polymerase domain potentially facilitates DNA/RNA synthesis and division, thereby benefiting cell growth and function^42^. In contrast, *nlrc3 card 15-like* serves as a negative regulator of bacterial/viral infections and the production of proinflammatory cytokines^43, 44^. The reduced expression of *nlrc3 card 15-like* in the white variety may help maintain higher innate immune levels. Moreover, the elevated expression of *nod2*, which promotes the formation of *blrp1-asc-caspase 1* inflammasomes^45^, in the spleen further supports the negative regulation of *nlrc3 card 15-like*. This observation may explain the smaller spleen index but stable immune function observed in the white variety. Hence, these three genes, namely *pcdhf4*, *pol-like*, and *nlrc3 card 15-like*, are likely to contribute to the growth and development advantage observed in the white variety. Moreover, they hold potential as molecular markers for future breed optimization.

## Material and method

### Experimental fish

The experiment was conducted in an authentic cultural environment, where broodstocks of white *C. argus* and wild ones are spawned and fertilized on a farm in Neijiang city, China. All fish were hatched through natural spawning, with a parent male: female ratio of 1:1. Light and water temperature changed periodically due to the open-air environment. Throughout the experiment, *C. argus* was provided with commercial feed containing the following components (%): Crude protein ≥ 48, crude fiber ≤ 4, crude ash ≤ 18, crude fat ≥ 6, water ≥ 12, sodium chloride 0.4–5.0, lysine ≥ 2.6, calcium 0.5–5.0, total phosphorus 0.5–5.0. The feeding regimen involved giving 5% of the fish’s weight before reaching 50 g (1 time/4 h) and 3% of the fish’s weight after reaching 50 g (1 time/day, feeding time was 6:00–7:00 a.m.).

For this study, ten individuals from two varieties of *C. argus*, at 120 dpf and 150 dpf, were randomly selected as subjects to compare their growth characteristics. Before conducting anatomical examinations, a thorough anesthesia protocol was administered to all fish specimens, following local standard practices. This procedure involved immersing the fish in a solution containing 100 mg/L of MS-222 until they exhibited no response upon manual tail manipulation, signifying a state of profound anesthesia. Following the documentation of their weight and length measurements, the fish were subsequently subjected to euthanasia via caudal venipuncture. Organ indexes (organ weight/body weight × 100%) were determined by individually weighing the liver, spleen, trunk kidney, heart, gill, and skeletal muscle. These organs were then meticulously fixed in 10% neutral formalin for histological examination. Additionally, liver tissue, spleen, and intestinal content samples were collected and rapidly fixed in liquid nitrogen, followed by storage at − 80 °C. These samples will be used for gene expression analysis and microbial community detection.

### Ethical approval

A statement to confirm that all animal handling procedures were approved by the Institutional Animal Care and Use Committee (IACUC) of the Sichuan Agricultural University, following the recommendations in the ARRIVE guidelines. At the same time, all methods were carried out in accordance with relevant guidelines and regulations.

### Histological examination and evaluation

Histological analysis was performed on ten individuals from each variety. Samples of the liver, spleen, trunk kidney, intestine, and skeletal muscles at the caudal peduncle were collected and subsequently fixed in a 10% neutral formalin fixative. The fixed tissues were then trimmed into cassettes, subjected to dehydration in graded ethanol solutions, cleared in xylene, embedded in paraffin wax. Following this, four-micrometer sections were obtained and stained with hematoxylin and eosin (H&E) for examination under an Eclipse 50i light microscope (Nikon, Tokyo, Japan).

To ensure a comprehensive evaluation, five visual fields were randomly selected for image collection in each section. All images were appropriately labeled with identifiers and organized for analysis. A blind evaluation of histologic measurement was conducted using Image Processing and Analysis in Java (Image J) 1.8.0 (National Institutes of Health, USA). The relevant parameters for each fish were determined based on the average of the visible fields.

### Intestinal microbial diversity analysis

In this study, a total of nine fish were selected from each variety for 16S-seq analysis of their intestinal microbiota. Certainly, as mentioned above, all fish were subjected to anesthesia and euthanasia procedures using the MS-222 compound in conjunction with caudal venipuncture. The intestine was carefully excised and rinsed several times with 0.65% sterile saline. Subsequently, the intestinal contents from the midgut to hindgut of three fish were pooled together to form one composite sample. Genomic DNA was extracted from the combined intestinal contents using a bacterial DNA isolation kit (Foregene Co., Ltd., China) following the manufacturer's instructions. The integrity of the extracted genomic DNA was verified by 1% agarose gel electrophoresis.

For PCR amplification, a forward primer (338F: 5′-ACTCCTACGGGAGGCAGCAG-3′) and a reverse primer (806R: 5′-GGACTACHVGGGTWTCTAAT-3′) were employed. The PCR reactions comprised a 3-min initial denaturation step at 95 °C, followed by 27 amplification cycles at 95 °C for 30 s, 55 °C for 30 s, and 72 °C for 45 s, with a final extension step at 72 °C for 10 min. The PCR product were examined by 2% agarose gel electrophoresis, and subsequently purified using the AxyPrep DNA Gel Extraction Kit (Axygen, USA). The DNA concentration of the purified products was determined using the QuantiFluor™-ST Blue Fluorescence System (Promega, China), prior to next-generation sequencing.

Sequencing library was constructed for the V3-V4 amplicons, and paired-end (PE) sequencing was conducted using the MiSeq System (Illumina, USA). To obtain full-length sequences, paired reads were merged based on their overlapping relationship using Flash. Prior to analysis, the dataset was subjected to exclusion criteria: sequences shorter than 50 bp, those with < 10 bp in the libraries, and sequences with ambiguous nucleotides constituting over 20% of the sequence were removed from the dataset. The remaining sequences were clustered into operational taxonomic units (OTUs) using Uparse 7.0.1090 with a similarity cutoff of 97%^46^. Subsequently, OTU analysis was performed using Usearch 7.0 (http://drive5.com/uparse/).

For species classification, each OTU was compared against the SILVA 16S rRNA database (https://www.arb-silva.de/) using BLAST analysis. Species composition analysis was carried out using Circos. Species that exhibited a relative abundance rate less than 0.01 in all samples were categorized as “others.” To evaluate differences between sample groups, *t*-tests (*P* < 0.05) were conducted using SPSS 22.0.

### Transcriptomics (RNA-seq) analysis

RNA-seq analysis was conducted to investigate the gene expression profiles in two varieties of *C. argus*, utilizing liver and spleen tissues of three individuals at 150 dpf. Each organ was subjected to three replicates. The process began with the extraction of total RNA from the samples, followed by assessment of its concentration, purity, and integrity using Nanodrop 2000 (Thermo, Waltham, USA) and Agilent Bioanalyzer 2100 (Agilent, Palo Alto, USA). Magnetic beads with Oligo (dT) were employed to enrich mRNA, which was then fragmented using a fragmentation buffer. Subsequently, cDNA fragments of 150–200 bp in length were selected and purified using the AMPure XP system (Beckman Coulter, Beverly, USA). To prepare the final library, 3 μL of USER Enzyme (NEB, USA) was used for a specific enzymatic process at 37 °C for 15 min, followed by 5 min at 95 °C prior to PCR. The PCR step involved Phusion High-Fidelity DNA polymerase, Universal PCR primers, and Index (X) Primer. The resulting PCR products were further purified (AMPure XP system) and evaluated for library quality using the Agilent Bioanalyzer 2100 system. The effective concentration of the library, quantified by qPCR (the effective concentration > 2 nM), determined the suitability for transcriptome sequencing on the Illumina Novaseq 6000 platform (Illumina, Santiago, USA).

The raw data in FASTQ format was initially processed through in-house perl scripts. The quality of the raw data was carefully evaluated, followed by removal of redundancy to obtain the unigenes. Clean reads were obtained after discarding subassemblies and low-quality sequences, which were then subjected to transcript assembly using Trinity 0.12.8^47^. Subsequently, these clean reads were aligned with the reference genome GCA_004786185.1 (https://www.ncbi.nlm.nih.gov/genome/?term=Channa+argus) using BOWTIE 2.4.1^48^ to generate the SAM/BAM file. The list of read counts was obtained through transcript clustering and quantification using CORSET 1.03. BOWTIE was further employed to compare fragments from each sample to transcripts, and the abundance information of each fragment was subjected to statistical analysis.

To gain insight into the functional significance of the obtained unigenes, we performed comparisons with various databases, including COG, GO, KEGG, KOG, Pfam, Swiss-prot, evolutionary genealogy of genes: Non-supervised Orthologous Groups (eggnog), and Ref-Seq non-redundant proteins (Nr) databases, using BLAST tool 40 with an e-value threshold of ≤ 1e−5^49^. The annotation information thus obtained enabled us to analyze the expression levels of differentially expressed genes (DEGs) using Fragments/KB/Million reads (FPKM). DESeq2 algorithms^50^ were employed to identify a subset of differentially expressed genes (DEGs), with *P*-value corrected by multiple hypothesis tests using the BH method (screening threshold: FDR ≤ 0.05, Abs (log_2_ Fold Change) ≥ 2)^50^. The annotation information of DEGs was then used to extract relevant data regarding DEGs for subsequent KEGG significant enrichment analysis^51^, aimed at determining the regulatory pathways involved.

### Statistical analysis

The findings are presented as the mean value along with its corresponding standard deviation. To ascertain the significance of dissimilarities, variance analysis was conducted. The data were subjected to ANOVA, and subsequently, the Duncan test was employed to assess the statistical significance of inter-group variations (SPSS v.22.0, IBM Corp., Armonk, New York, USA). Significance was defined as a *P*-value less than 0.05, while high significance was established for a *P*-value less than 0.01.

### Supplementary Information

Supplementary Figures.

Supplementary Table 1.

## Data Availability

The datasets underlying the findings presented in this article have been included within the manuscript. Additionally, the raw data utilized for transcriptome analysis can be accessed from the GenBank repository (NCBI: SRR23938328-SRR23938339). e.g., https://www.ncbi.nlm.nih.gov/sra/?term=SRR23938338.
