# Supplementary material for: Effects of leucism on organ development and molecular mechanisms in Northern snakehead (Channa argus) beyond pigmentation alterations
Source: Sci Rep. 2023 Nov 11;13:19689. doi: 10.1038/s41598-023-46608-9 (PMC10640583; doi:10.1038/s41598-023-46608-9)
Supplement: Supplementary file 1 — Supplementary Figures. [file 41598_2023_46608_MOESM1_ESM.docx]

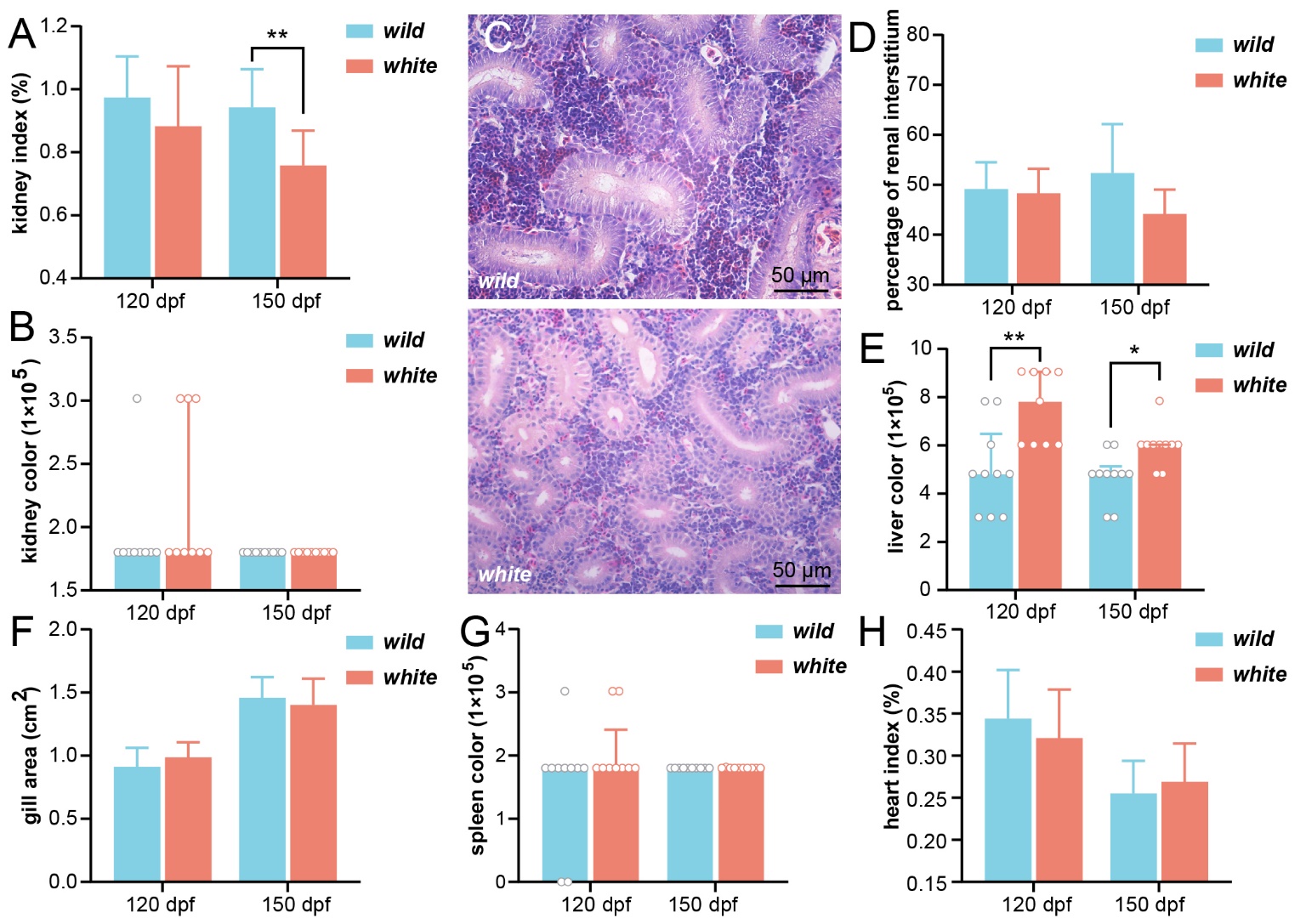


Fig. S1 Comparison of organ development in white and wild *C. argus*

A, F, & H showed the kidney index, gill area, and heart index in White and wild *C. argus*, respectively. B, E, & G showed the color of kidney, liver and spleen in White and wild *C. argus*, respectively. The color of organ were recognized by a Image color recognition tool in website ChinaZ (<https://tool.chinaz.com/Tools/img>), and the color was represented by a six-digit code, with a larger number indicating a lighter color, e.g., Black represents #000000 and white represents #ffffff. C showed the histology of kidney (H&E×400) in White and wild *C. argus*. D showed the histologic evaluation of kidney by Image J analysis in White and wild *C. argus*. *, or ** represents a significant difference (*P*<0. 05) or highly significant difference (*P*<0. 01) between two varieties.


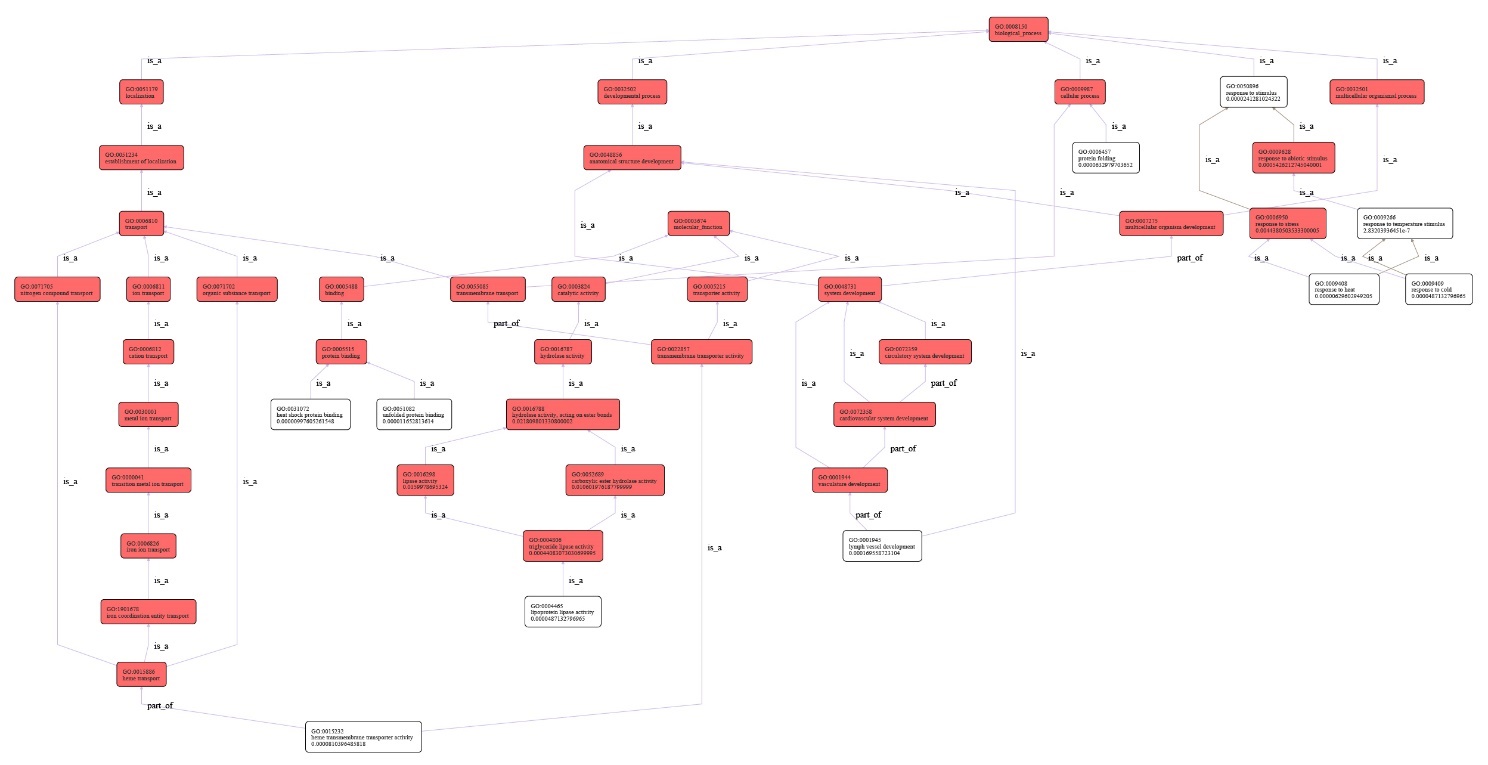


Fig. S2 The top GO terms of the DEGs in the liver.


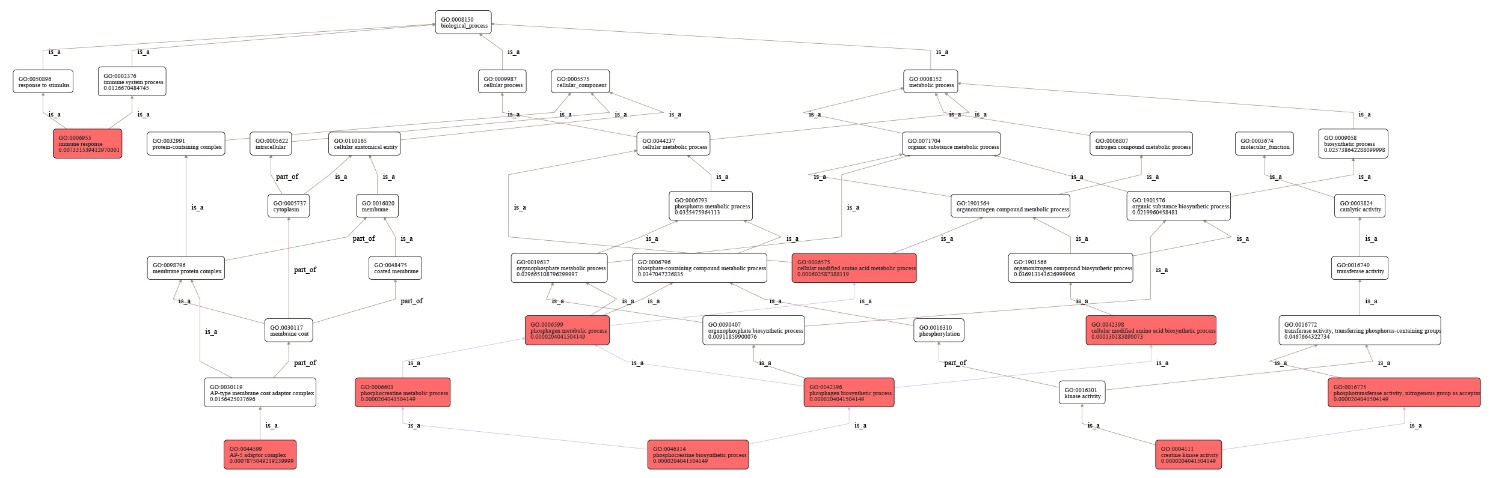


Fig. S3 The top GO terms of the DEGs in the spleen.
